# Synchrotron imaging reveals bone healing and remodelling strategies in extinct and extant vertebrates

**DOI:** 10.1098/rsif.2014.0277

**Published:** 2014-07-06

**Authors:** Jennifer Anné, Nicholas P. Edwards, Roy A. Wogelius, Allison R. Tumarkin-Deratzian, William I. Sellers, Arjen van Veelen, Uwe Bergmann, Dimosthenis Sokaras, Roberto Alonso-Mori, Konstantin Ignatyev, Victoria M. Egerton, Phillip L. Manning

**Affiliations:** 1 School of Earth, Atmospheric and Environmental Sciences, University of Manchester, Williamson Building, Oxford Road, Manchester M13 9PL, UK; 2 Department of Earth and Environmental Science, Temple University, Philadelphia, PA 19122, USA; 3 Faculty of Life Sciences, University of Manchester, Manchester M13 9PL, UK; 4 SLAC National Accelerator Laboratory, Linac Coherent Light Source, Menlo Park, CA 94025, USA; 5 SLAC National Accelerator Laboratory, Stanford Synchrotron Radiation Lightsource, Menlo Park, CA 94025, USA; 6 Diamond Light Source, Didcot OX11 0DE, UK

**Keywords:** SRS–XRF, archosaur, histology, bone, fracture healing

## Abstract

Current understanding of bone healing and remodelling strategies in vertebrates has traditionally relied on morphological observations through the histological analysis of thin sections. However, chemical analysis may also be used in such interpretations, as different elements are known to be absorbed and used by bone for different physiological purposes such as growth and healing. These chemical signatures are beyond the detection limit of most laboratory-based analytical techniques (e.g. scanning electron microscopy). However, synchrotron rapid scanning–X-ray fluorescence (SRS–XRF) is an elemental mapping technique that uniquely combines high sensitivity (ppm), excellent sample resolution (20–100 µm) and the ability to scan large specimens (decimetre scale) approximately 3000 times faster than other mapping techniques. Here, we use SRS–XRF combined with microfocus elemental mapping (2–20 µm) to determine the distribution and concentration of trace elements within pathological and normal bone of both extant and extinct archosaurs (*Cathartes aura* and *Allosaurus fragilis*). Results reveal discrete chemical inventories within different bone tissue types and preservation modes. Chemical inventories also revealed detail of histological features not observable in thin section, including fine structures within the interface between pathological and normal bone as well as woven texture within pathological tissue.

## 1. Introduction

The morphological characterization and description (histological analysis) of extant bone is used in the comparative interpretation of extinct vertebrates often providing evidence for physiological properties such as age, reproduction, healing strategies and metabolic rates [1–5]. Histological analysis of fossil material can also aid in determining taphonomic alteration through the identification of crystal growth replacement within bone tissue [5–7]. However, in order to obtain a more complete understanding of what original details remain in fossil bone, it is important to distinguish between biologically derived constituents (endogenous material) and taphonomic artefacts added during fossilization of extinct organisms.

Although structural morphology can be preserved in a fossil despite mineral replacement, the current paradigm suggests that the original biochemical composition has been lost [6–10], with some exceptions concerning the high durability of enamel [9–13]. Recent studies, however, have shown that endogenous chemistry can survive in even the most fragile of tissues such as rare fossils of skin [14]. Many conventional techniques have mapped soft tissue preservation in fossils and determined that these preserved tissues are chemically different from the matrix, i.e. not just impressions [14–20]. If such delicate tissues can preserve endogenous biomarkers, then it is likely that hard tissues such as bone may also contain endogenous trace elements that are critical to hard tissue mechanical properties and physiology [21].

The chemistry of original bone apatite is often defined as hydroxyapatite, but is more complex owing to the multiple substitutions that can occur within the apatite structure [8,22,23]. The ability of bone to substitute a wide range of elements makes it an important sink for trace elements essential to various physiological functions, such as fracture healing [1,24–27]. Because the rate and strategy of fracture healing are heavily dependent on physiological factors such as metabolism, nutritional status and immune response [1,21,28–30], it may be possible to use fracture healing as means to interpret physiology in extinct and extant taxa.

To fully understand the biosynthetic pathways employed by vertebrates in bone maintenance and repair, knowledge of the trace element inventory of bone is crucial. Specific trace elements such as copper, zinc and strontium have been shown to have profound impacts on bone physiology [31–35]. These elements are elevated around an affected area during the distinct stages of fracture healing allowing for the possibility of using trace elements as biomarkers for diagnosing the degree of callus maturation. Zinc has been found to stimulate bone formation and is usually correlated with areas of active ossification [31,32,36,37]. The addition of extraneous strontium increases osteoblastic activity and inhibits osteoclast proliferation and differentiation [31,32] resulting in an increase in bone deposition. This has made strontium the most studied of the trace elements related to fracture healing owing to its potential as a drug (strontium renelate, SR) to help treat osteoporosis [31,32]. Unlike zinc and strontium, copper is important for the organic constituent of bone as it is essential in the formation of cross-links found in collagen [35,38], which prevent the unravelling of the collagen protein chains. However, the distribution and dilute concentrations of key trace elements that are crucial to maintaining healthy bone are difficult to map within discrete biological structures. The advent of synchrotron-based imaging of biological and palaeontological samples is helping address these issues [17–20].

Synchrotron rapid scanning–X-ray fluorescence (SRS–XRF) combines high sensitivity (concentrations of ppm) with excellent sample resolution (from 20 to 100 µm) and the ability to scan large specimens (100 × 100 × 30 cm and up to 25 kg) almost 3000 times faster than other acquisition techniques [16–20]. SRS–XRF has been optimized to allow multi-element mapping and can also be combined with X-ray absorption spectroscopy (XAS) [17–20,39,40]. This allows for the determination of the atomic coordination of a particular element (how it is bound to other elements). This is important when determining whether or not an element is organic or inorganic in origin and thus whether it should be attributed to the tissue or to taphonomic processes [17–20].

Previous studies have used SRS–XRF coupled with XAS to examine iconic fossils such as *Archaeopteryx* [17] as well as exceptionally preserved fossils from the Green River Formation, USA [14,18,20]. In these studies, SRS–XRF of fossils has revealed biological structures that cannot be observed in visible light as well as the fractionation of elements within discrete biological structures that can be compared with comparable tissue in living organisms. Such studies have led to the identification of specific elemental biomarkers such as eumelanin [19].

Given the propensity for trace metals mediating crucial enzymatic reactions during syntheses of extant tissues, it is important to identify if similar controls during bone genesis can be identified in fossil material. In this study, we use SRS–XRF coupled with XAS to determine the elemental composition and variation in concentrations in pathological and normal bone from both an extant and extinct archosaur (*Cathartes aura* and *Allosaurus fragilis*). Theropods are a logical study group as they are more closely related to the most diverse group of extant archosaurs, the birds. The extant avian homologue potentially provides great insights into the biology, biochemistry and physiology of their extinct dinosaur ancestors.

## 2. Methods

The techniques used in this study included both standard morphological and histological approaches to the preparation and analysis of extant and extinct samples.

### 2.1. Specimens

A distal pedal phalanx (digit III-3) of the Late Jurassic theropod dinosaur *A. fragilis* (UMNH 6282; Morrison Formation, Cleveland-Lloyd Quarry, UT, USA) was used in this study. This specimen exhibits a smooth callus on the external dorsal surface.

A proximal-most phalanx of the lateral toe (digit IV-1) of an extant turkey vulture, *C. aura* (DMNH 83356), was used for comparison. The external surface of the plantar–distal end of the phalanx is almost entirely covered with reactive bone, thereby giving a frothy appearance.

### 2.2. Thin section preparation

All histological analysis was performed using the thin section facilities of the Department of Earth and Environmental Science at Temple University (PA, USA). Thin section specimens from the *A. fragilis* and *C. aura* specimens were prepared by cutting along the paramedian plane through the callus for histological analysis. To prevent fracturing and flaking of the fossil, the specimen was vacuum impregnated with Paleobond penetrate stabilizer (PB002) before grinding billets down to the desired thicknesses of 100–120 µm [41]. Thin sections were viewed in both plane and cross-polarized light using a Nikon ECLIPSE E600 POL microscope and ACT-1 software. Histological data were compared with elemental data obtained from SRS–XRF to identify any associations between bone tissue types and specific elemental signatures.

### 2.3. Elemental mapping

Both *A. fragilis* and *C. aura* were analysed at the Stanford Synchrotron Radiation Lightsource (SSRL, CA, USA) on wiggler beamline 6-2. This beamline is one of the few facilities that can provide large-scale SRS–XRF, which allows for the rapid mapping of large sample surface areas and is preferred when first assessing the overall elemental distribution of larger specimens. The *A. fragilis* section was also analysed at the microfocus beamline, I-18, at the Diamond Light Source (DLS, Oxford, UK). The area of interest that can be imaged on this beamline provides a smaller, but higher resolution scan than the facilities of SSRL beamline 6-2, which allows us to concentrate on areas of interest such as the interface between pathological and normal bone.

#### 2.3.1. Stanford Synchrotron Radiation Lightsource

Specimens were mounted on an *x–y–z* motor-controlled stage and moved in a raster pattern relative to the fixed incident beam [17–19]. Experiments consisted of an incident beam energy either at 13.5 keV for heavier (high-*Z*) elements (Ca and heavier) and or at 3.15 keV for lighter (low-*Z*) elements (Ca and lighter). Flux was calculated to be between 10^10^ and 10^11^ photons s^−1^ at high *Z*, and approximately 10^9^ photons s^−1^ at low *Z*. Beam diameters of 50 µm were used for high-*Z* elements and 100 µm for low-*Z* scans. For high-*Z* maps, specimens were analysed under ambient conditions and aligned at a fixed incident angle of 45° relative to the beam with the single element drift detector (Vortex) set at a 90° scattering angle to the incident beam. For low-*Z* maps, the specimen was placed in a helium-purged sample chamber, and the scattering angle was changed to 160° to minimize signal loss. The helium atmosphere is necessary to avoid X-ray absorption and scattering effects of air that can attenuate the beam at lower incident beam energies [17].

XRF energies of interest were assigned to the detector to capture specific emission energies (up to 16 simultaneous element windows) simultaneously during mapping. To obtain rapid scanning, a full energy-dispersive X-ray spectrum is not recorded for each pixel [20]. The element windows are selected by collecting a raw energy-dispersive X-ray spectrum from 10 to 20 raster lines over an area of the map containing the majority of the different materials present in the sample (e.g. matrix, soft tissues and hard tissues) [17–20]. The averaged spectrum is used to assign the elemental windows (e.g. CaKα, ZnKα, BaLα, etc.), so that all elements are chosen based on their dominance within the energy-dispersive X-ray spectrum (electronic supplementary material). The imaging software ImageJ [42] was used to calculate spatial correlations from maps using the Image CorrelationJ plugin [43].

To quantify the concentrations of elements within a sample, point analyses were selected by locating an area of interest within the scan, driving the stage to the point coordinates and collecting a full energy spectrum for 50 s (electronic supplementary material, figures S1 and S2). Three point analyses for high-*Z* (Ca and heavier) and two for low-*Z* (Ca and lighter) scans were taken at each area of interest and averaged to account for sample heterogeneity (electronic supplementary material, figures S1 and S2). Only two scans were taken at low *Z* owing to the constraint of experimental time available. The software package PyMCA [44] was used to fit point spectra based on the raw energy-dispersive X-ray spectroscopy files and from the experimental parameters using a Durango apatite standard of known element concentrations for calibration (electronic supplementary material) [17–20].

X-ray absorption near-edge structure (XANES) of the *Allosaurus* phalanx was conducted in fluorescence mode by scanning the incident beam energy through the sulfur K edge to determine sulfur speciation [17,19,20]. Calibration of the beam energy for XANES analyses was accomplished using a K_2_SO_4_ standard. XANES is particularly important to undertake on fossil samples given it helps identify the endogenous and exogenous facets of the sample. Thus, XANES was not performed on the modern bones, given that the endogeneity of the sample was not in question.

#### 2.3.2. Diamond Light Source

Scans were undertaken at the microfocus beamline I18 using a 6.5 µm diameter pinhole with an incident beam energy of 16.5 keV to pick up the SrKα emissions (flux 10^10^ to 10^11^ photons s^−1^). The sample was mounted on an *x–y–z* stage and rastered at a 45° angle to the incident X-ray beam with a single element drift detector (Vortex) set to 90° scattering angle. Windows are assigned to the detector post-data collection as a full spectrum is collected for each pixel. Maps were processed using the ROI tool in PyMCA [44]. Point analyses were selected from individual spectra within ROI maps.

The experimental set-up at DLS allows for the full extended X-ray absorption fine structure (EXAFS) to be performed. Zinc EXAFS of the *Allosaurus* material was conducted to determine whether the bonding coordination was comparable with that of hydroxyapatite (bone). EXAFS was fitted using the Demeter software package [45].

## 3. Results

### 3.1. *Allosaurus fragilis* (UMNH 6282)

**Figure 1.**
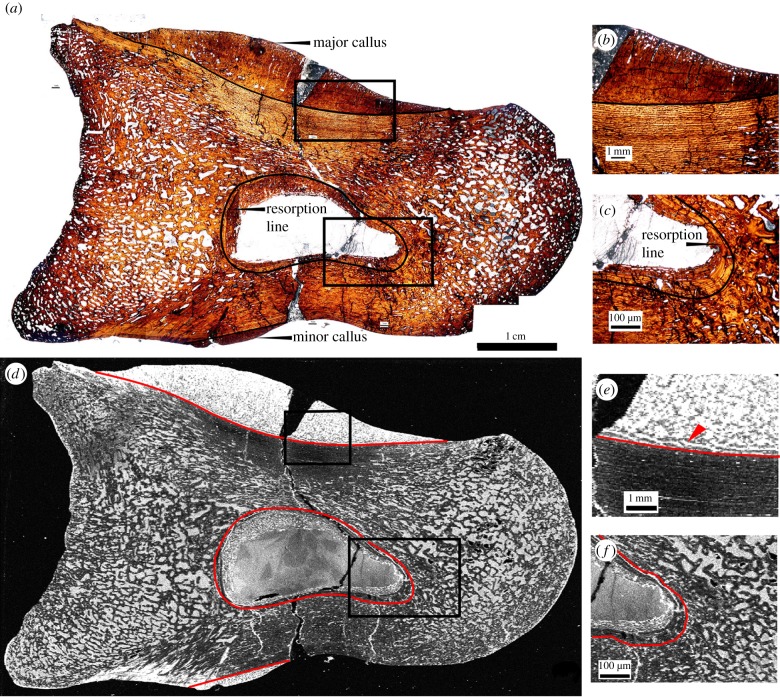
Thin section of *Allosaurus fragilis* (specimen UMNH 6282) pedal phalanx as seen in optical observation under plain polarized light (*a–c*) and in an elemental map of iron taken at the SSRL beamline 6-2 (*d–f*; grey scale relative image with white as high concentration, black as low). Calli are present on the dorsal (major callus) and ventral (minor callus) surfaces in both optical and elemental images. Photomicrographs (*b,c,e,f*) represent magnified views of areas of interests (boxed areas), including the major callus (*b,e*) and resorption cavity (*c,f*). Laminar orientation of bone tissue is observed in the map of iron around the interface of the major callus and normal cortical bone (*e*) that is not seen in thin section (*b*). The extent of resorption and woven bone infill of the medullary cavity are also enhanced in iron (*f*) compared with thin section (*c*).

**Figure 2.**
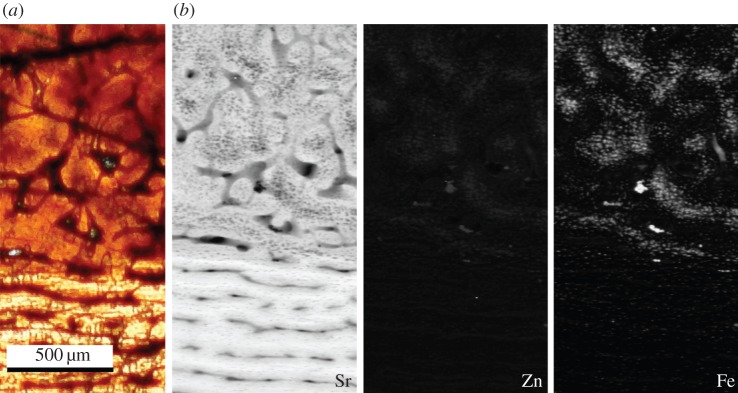
(*a*) Histological section as observed under plane polarized light and (*b*) false colour elemental maps of strontium, zinc and iron at the boundary between normal and pathological bone of the major callus in specimen UMNH 6282. The map of strontium highlights the woven texture of the callus, boundary of the bone tissues and laminar orientation of the normal bone. Zinc and iron are found mainly within the woven bone tissue of the callus.

Two smooth calli are identified optically by changes in bone tissue orientation as observed under plane polarized light (figure 1): a large callus on the dorsal surface (major callus) and a smaller callus on the ventral surface. A post-mortem crack is present running from the distal-to-ventral surfaces, which appears as a void in the map of iron. In figure 1*a,c*, the interface between the pathological and normal tissue is labelled as a black (figure 1*a,b*) or red (figure 1*d,e*) line. The major callus is highly remodelled as shown by the maturity of the compacted cancellous tissue of the callus seen in both the optical observation of the histology (figures 1*b* and 2*a*) and in the map of iron and strontium (figures 1*e* and 2*b*, respectively). A medullary cavity is present in the centre of the phalanx (figure 1*a,c,d,f*). The furthest extent of resorption caused by the medullary cavity formation is marked by a resorption line, seen optically as a dark line surrounding the cavity (figure 1*a,c*; outlined in black) and as a bright white region (elevated concentration) in the map of iron (figure 1*d,f*; outlined in red). Between the lining of lamellar bone and the cavity void is a ring of compact cancellous bone of irregular thickness, which represents endosteal pathological bone deposition (figure 1*a,c,d,f*).

Elemental maps of iron (figures 1*d–f* and 2) and zinc (figure 2) show that chemistry is not uniformly distributed, but correlated with distinct bone tissue types, revealing histological details not observable via optical microscopy (histological thin section). Many of these details are affiliated with tissues seen in the calli and medullary cavity. Structures highlighted include laminar orientation of bone tissue located near the callus/normal bone interface in the medial section of the major callus (figure 1*d* indicated by a red arrow; figure 2*b*, respectively), the resorption line of the said cavity (figure 1*c,f*) and woven bone textures in pathological tissues (figures 1 and 2). The distribution of strontium and copper (the other elements known to strongly impact bone physiology) does not display differential uptake between tissue types.

**Figure 3.**
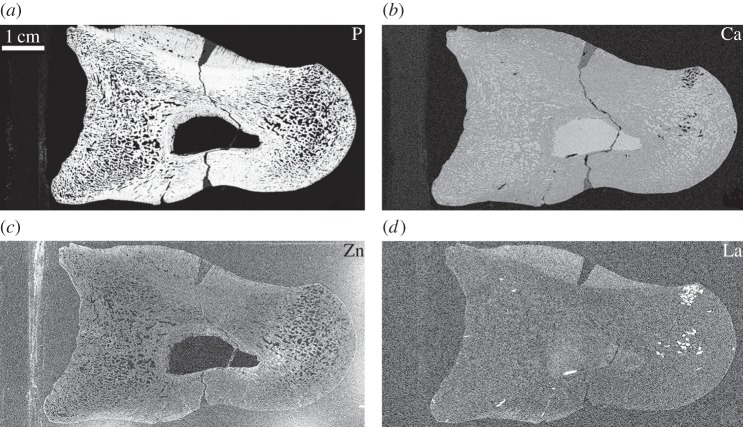
Elemental maps of phosphorus (*a*), calcium (*b*), zinc (*c*) and lanthanum (*d*) in UMNH 6282 taken at the SSRL beamline 6-2. Phosphorus is concentrated within the original tissue (*a*), whereas calcium concentrations are uniform throughout the entire specimen excluding the glass mounting slide of UMNH 6282 (*b*). Lanthanum is enriched in the pathological tissues of the calli (*d*). Owing to spectral overlap, the map of lanthanum convolves both lanthanum and barium. Lanthanum was distinguished from barium using PyMCA analysis [44].

Calcium concentrations are relatively uniform between bone tissue and the mineral infill, with concentrations comparable to those of fossil bone and calcite (figure 3*b* and table 1); however, phosphorus (figure 3*a*) is concentrated only within the fossil bone tissue. At the millimetre scale, zinc appears to be distributed evenly throughout the fossil bone (figure 3*c*); however, finer resolution scans of the pathological/normal bone interface reveal a strong distribution correlation between zinc and the woven pathological tissues of the main callus (figure 2).

**Table 1.** XRF point analysis concentrations taken at the SSRL of different bone tissue types and mounting slide of UMNH 6282 and the Durango standard. Concentrations are given in ppm or weight per cent (wt%). The margin of error is approximately 10% of the absolute value. This is a conservative error value.

	epoxy/glass	Durango standard	major callus (dorsal)	callus/cortical edge	cortical bone	medullary cavity	minor callus (ventral)	possible HREE
high *Z*								
Ca	1.68 (wt%)	38.2 (wt%)	32.89 (wt%)	34.06 (wt%)	37.50 (wt%)	31.61 (wt%)	30.35 (wt%)	14.20 (wt%)
Mn	289	—	5587	2195	965	4270	4861	525
Fe	428	583.4	2.11 (wt%)	8620	2190	1.30 (wt%)	2.12 (wt%)	2127
Cu	4	—	18	30	20	11	25	12
Zn	45	32.26	69	57	62	62	118	41
Ba	469	0	0	117	0	0	696	28.25 (wt%)
La	0	5094	1107	1059	630	1079	749	0
Ce	0	7852	2611	3041	2629	914	2402	1466
low *Z*								
Si	4907	1021	1.27 (wt%)	—	2292	1.34 (wt%)	1.67 (wt%)	6734
P	856	18.17	17.99 (wt%)	—	32.97 (wt%)	14.95 (wt%)	29.32 (wt%)	16.11 (wt%)
S	1925	0	1.28 (wt%)	—	2.23 (wt%)	1.16 (wt%)	1.78 (wt%)	9.93 (wt%)

Owing to the close proximity of the barium and lanthanum L-emission lines, these elements cannot be clearly windowed for the purposes of mapping, and therefore the map of lanthanum (figure 3*d*) inherently incorporates some signal from barium L-emission. However, processing the point analyses through PyMCA allowed for the identification and quantification of these two elements (electronic supplementary material). Lanthanum is concentrated mainly within the pathological tissue of the major dorsal callus with respect to all other bone tissues and the calcite infill of the fossil (figure 3*d*). All other areas of high concentration (bright spots) represent high concentrations of barium with respect to the entire thin section mount (table 1).

**Figure 4.**
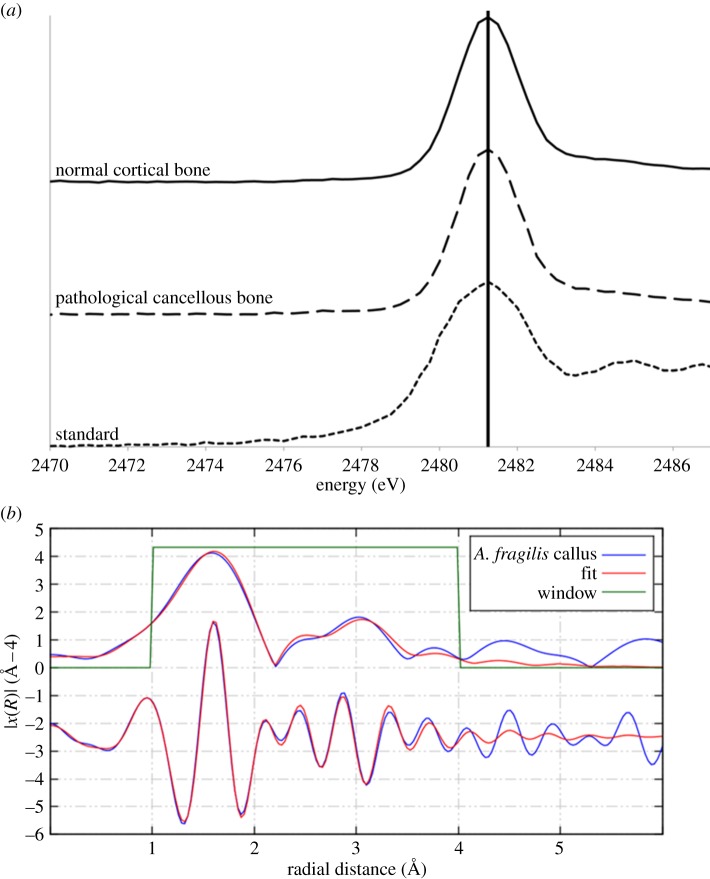
(*a*) XANES spectra for normal cortical bone taken from the dorsal surface and pathological bone taken from the main callus of UMNH 6282 compared with a sulfate standard. The peak for the sulfur species sulfate is marked [45]. (*b*) EXAFS spectrum for zinc taken from the main callus of UMNH 6282 revealing a tetrahedral coordination with phosphate.

XAS of UMNH 6282 revealed sulfur speciation to be inorganic sulfate [46] (figure 4*a*), and zinc to be in a tetrahedral coordination with four oxygens within phosphate (figure 4*b* and the electronic supplementary material).

### 3.2. *Cathartes aura* (DMNH 83356)

**Figure 5.**
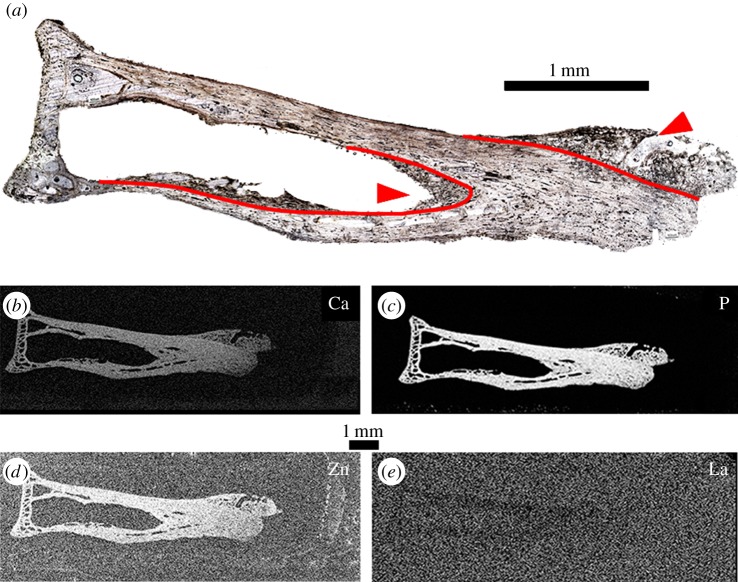
Thin section of DMNH 83356 as seen in optical observation under plane polarized light (*a*), calcium (*b*), phosphorus (*c*), zinc (*d*) and lanthanum (*e*) taken at SSRL (beamline 6-2). Woven pathological bone growth is present along both the endosteal surface of the mid-shaft and the distal articulation surface (arrowheads). The interface between pathological growth and the normal bone tissue outline is more distinct in elemental maps compared with optical observation (outlined in red), especially in determining the extent of pathological growth along the endosteal surface (*b–d*). Lanthanum is below detection limit within the bone tissue, which is expected in modern samples (*e*).

Cancellous bone growths are observed under plane polarized light within the endosteal surface of the mid-shaft and on the distal articular surface (figure 5*a*). Pathological bone consists of very poorly remodelled woven cancellous bone radiating from the original bone outline (figure 5*a*, pathological/normal bone interface marked in red). Pathological tissue was identified optically by the woven texture observed as the specimen is rotated in cross-polarized light. Elemental maps of calcium (figure 5*b*), phosphorus (figure 5*c*) and zinc (figure 5*d*) provide clear distinctions between the normal bone contour and the pathological bone growth, which is difficult to discern under optical observation, especially along the endosteal surface. Zinc and calcium concentrations are comparable to those found in other modern archosaurs [6] (table 2). Lanthanum is below the detection limit within the bone tissue (figure 5*e*), as expected for modern samples [7].

**Table 2.** High atomic weight element concentrations (Ca and heavier) in bone tissues and mounting slide of DMNH 83356 and the Durango standard. Concentrations are given in ppm or weight per cent (wt%). Error for heavy elements is approximately 10% of the absolute value. This is a conservative error value.

	epoxy/glass	Durango standard	pathological cancellous	normal cortical
Ca	1.72 (wt%)	38.2 (wt%)	30.64 (wt%)	25.86 (wt%)
Fe	392	583.4	212	167
Cu	2.44	—	11.20	40.30
Zn	43.30	32.26	198.47	221.13
As	0.37 (wt%)	870	0.27 (wt%)	0.21 (wt%)

## 4. Discussion

The ability to distinguish fine details in the interpretation of pathological bone tissue is essential for physiological interpretations of vertebrates. Optical histological analysis of fossilized tissues is strongly dependent on the quality of preservation of microstructures, which may be lost even within well-preserved fossils exhibiting exceptional gross morphology. In this study, large-scale high-resolution elemental mapping proved to be an effective tool in the interpretation of pathological healing strategies and histories within fossils where taphonomic processes may obscure fine histological features. Furthermore, elemental mapping proves to be a more powerful tool for discerning fine tissue features in extant material that may be obscured owing to sample preparation or size.

In *A. fragilis*, the discernibility of the morphological features from the pathological tissue of the major callus and endosteal bone growth within the medullary cavity was enhanced when compared with optical thin section observations. This includes details of the woven bone texture within the pathological tissue, as well as enhancement of the resorption line of the medullary cavity, which indicates the farthest extension of bone resorption. The laminar orientation of bone tissue observed in iron between the main callus and normal cortical bone indicates the areas of resorption and remodelling of the callus not seen in optical observation [14].

In *C. aura*, elemental maps of calcium, phosphorus and zinc highlight the interface between normal and pathological tissues by providing a more accurate means to distinguish between pathological bone growth and the normal bone tissue outline, especially at the distal pathological/normal interface along the endosteal surface (figure 5). Elemental mapping thus provides additional morphological data compared with traditional optical thin section analysis, thereby allowing for the identification of discrete morphological features in both extant and extinct specimens.

Elemental maps revealed discrete elemental inventories unique to specific biological pathways within distinct tissue types, particularly in zinc. Zinc is usually correlated within areas of ossification, especially within areas of osteoid mineralization such as within osteons [31,32,36,37]. The distribution of zinc was constrained within the woven tissue of the fracture callus in *A. fragilis*, a tissue type expected to be undergoing active ossification at time of death (figure 2). Quantification of zinc is slightly depleted (57–69 ppm) compared with modern archosaurs (100–250 ppm) [6,47] with the exception of the minor callus (118 ppm). This may suggest that at least a small portion of the original zinc has been removed by geological processes. If zinc has been removed from the fossil over time, then we should see the lowest concentrations of zinc in the more porous bone tissue (such as the calli and resorption cavity) and the outer edges of the bone as observed with the rare earth element (REE) concentrations (lanthanum). However, zinc concentrations remain fairly uniform throughout the entire specimen.

Conversely, the addition of zinc through pore water should also be taken into consideration, especially given the close association between the distribution of zinc and iron within the fracture callus as seen in figure 2. However, if zinc was introduced to the system, then we should see much higher concentrations than what is to be expected from modern bone (over 200 ppm). This trend is seen in iron and lanthanum concentration profiles, suggesting an exogenous source to these elements. In addition, zinc EXAFS indicates that zinc is bound to four oxygen atoms in a tetrahedral coordination within phosphate, which is the coordination observed in hydroxyapatite [48]. If zinc had been added exogenously, then we would expect it to be coordinated with either the inorganic sulfate or an iron oxide. The zinc occupies the same positions as iron within the pathological bone tissue, but is not bound to the iron, suggesting that the zinc is endogenous.

In *C. aura*, zinc concentrations are elevated in the normal cortical tissue compared with the pathological cancellous tissue (table 2). Because we did not have access to this specimen at the microfocus beamline at DLS, we cannot say whether this discrepancy in concentrations is due to the large size of the pinhole at SSRL (50–100 µm), which is too large to distinguish between some of the finer histological features (i.e. cancellous struts). However, the concentrations of trace elements within *C. aura* calculated within this study are comparable with both other modern archosaurs and the *A. fragilis* specimen for exogenous elements such as calcium and zinc [6] (tables 1 and 2). We therefore suggest that zinc should be pursued as a possible biomarker for active ossification, and will be the subject of future work on both extant and extinct taxa. We were unable to discern differential uptake of strontium or copper between bone tissue types. Although copper concentrations appear to be slightly elevated within some tissue types, the difference in concentration is within the error of our quantification calculations.

Elemental mapping was also useful for determining the taphonomic history of a specimen through the diffusion patterns of elements as well as the quantification of elemental concentrations. The pattern and rate of elemental incorporation affects the rate of fossilization, which affects the probability of preserved original material through the degree of diagenetic alteration [9–12]. In *A. fragilis*, REE concentrations appear to follow simple diffusion patterns seen in most taphonomic studies where concentrations of REE decrease moving medially into the bone [7,10,12,49]. However, differences are observed between the pathological bone of the calli and the cancellous bone of the articular surfaces with concentrations of the light REE (lanthanum) elevated within the pathological tissue compared with all normal bone tissues. Because the common factors that affect REE uptake are similar for both tissue types (bone porosity, location to surface of the bone, size of element), another tissue property must be responsible. One possibility is that the calli were still soft (cartilage present) at least at the outer surface of the callus. Soft tissues would degrade quickly, exposing the porous pathological bone faster than the normal cancellous bone, which is surrounded by the dense lamellar bone of the outer circumferential layer. If this callus is the result of fracture healing, then the presence of cartilage in a callus that histologically appears to be very well remodelled would indicate a more reptilian style of callus, which is more cartilaginous than mammalian calli in later stages of remodelling [28–30].

The identification of cartilage is important for a number of biological interpretations, including maturity (age), size (length and height), biomechanics and healing strategies. Currently, identification of cartilage in the fossil record is based on morphological data [1], and a definitive trace metal biomarker for cartilage is yet to be identified. Morphological indicators of cartilage include attachment features such as the ‘pitted’ appearance of the articular ends of podial elements and the histological identification of calcified cartilage [1]. Unfortunately, fracture callus cartilage does not leave such morphological evidence on the surrounding bone. Therefore, future work on the effects of cartilage on REE uptake in modern and fossil bone is needed to test whether REE profiling may be helpful in the identification and quantification of cartilage in extinct organisms.

In the case of UMNH 6282, calcium concentrations are constant throughout the specimen, which suggests calcium remains immobile through the fossilization environment (table 1). This is not surprising as the lithologies of the Cleveland-Lloyd Dinosaur Quarry are highly calcareous, which would account for the calcite infill of the bone [50,51] and the stability of endogenous bone calcium. Concentrations of iron are elevated compared with modern bone (wt% versus ppm) [6], suggesting an exogenous source (table 1). Sulfur was found to be present as inorganic sulfate rather than organic sulfur, suggesting that any original sulfur has been lost diagenetically. The incorporation of elemental data of the surrounding matrix would aid in determining the origin of the sulfur; however, this specimen had already been prepared before analysis. Furthermore, the heterogeneous nature of the site makes it difficult to collect a matrix sample for future analysis, because the sample may not be representative of the area from which the specimen was collected [50,51].

## 5. Conclusion

SRS–XRF elemental mapping provides a de novo approach to distinguish fine bone tissue chemistry and morphologies of extant and extinct specimens not observed in conventional histological thin section analysis. Many of these features occur at the boundaries between different tissue types, such as between pathological and normal bone, leading to more developed interpretations of healing strategies within archosaurs. Elemental maps also reveal a selective distribution of zinc within areas of possible active ossification in the pathological callus tissue of *A. fragilis*, suggesting a possible trace metal biomarker for the ossification process. This marks the first instance of a possible trace element biomarker for a biosynthetic pathway within fossilized bone tissue.

Future studies employing chemical analysis (SRS–XRF, Fourier transform infrared spectroscopy, etc.) are needed to resolve the identification and interpretation of trace metal biomarkers crucial to bone physiology. Coupled with the application of micro-computed tomography structural analysis, this approach could lead to the identification of bone healing strategies (callus architecture, protein pathways, speed of healing, etc.) used among extinct vertebrates.
